# The association between malaria parasitaemia, erythrocyte polymorphisms, malnutrition and anaemia in children less than 10 years in Senegal: a case control study

**DOI:** 10.1186/1756-0500-5-565

**Published:** 2012-10-11

**Authors:** Roger CK Tine, Magatte Ndiaye, Helle Holm Hansson, Cheikh T Ndour, Babacar Faye, Michael Alifrangis, K Sylla, Jean L Ndiaye, Pascal Magnussen, Ib C Bygbjerg, Oumar Gaye

**Affiliations:** 1 Service de Parasitologie, Faculté de Médecine et Pharmacie, Dakar, Sénégal; 2 Clinique des Maladies Infectieuses, Centre Hospitalier Universitaire de Fann, Dakar, Sénégal; 3 DBL - Centre for Health Research and Development, Faculty Health and Medical Sciences, University of Copenhagen, Copenhagen, Denmark; 4 Department of International Health, Immunology and Microbiology, Faculty of Health Sciences, University of Copenhagen, Copenhagen, Denmark

**Keywords:** Anaemia, Malaria, Haemoglobin disorders, Malnutrition, Children

## Abstract

**Background:**

Malaria and anaemia (Haemoglobin <11 g/dl) remain frequent in tropical regions and are closely associated. Although anaemia aetiologies are known to be multi-factorial, most studies in malaria endemic areas have been confined to analysis of possible associations between anaemia and individual factors such as malaria. A case control study involving children aged from 1 to 10 years was conducted to assess some assumed contributors to anaemia in the area of Bonconto Health post in Senegal.

**Methods:**

Study participants were randomly selected from a list of children who participated in a survey in December 2010. Children aged from 1 to 10 years with haemoglobin level below 11 g/dl represented cases (anaemic children). Control participants were eligible if of same age group and their haemoglobin level was >= 11 g/dl. For each participant, a physical examination was done and anthropometric data collected prior to a biological assessment which included: malaria parasitaemia infection, intestinal worm carriage, G6PD deficiency, sickle cell disorders, and alpha-talassaemia.

**Results:**

Three hundred and fifty two children < 10 years of age were enrolled (176 case and 176 controls). In a logistic regression analysis, anaemia was significantly associated with malaria parasitaemia (aOR=5.23, 95%CI[1.1-28.48]), sickle cell disorders (aOR=2.89, 95%CI[1,32-6.34]), alpha-thalassemia (aOR=1.82, 95%CI[1.2-3.35]), stunting (aOR=3.37, 95%CI[1.93-5.88], age ranged from 2 to 4 years (aOR=0.13, 95%CI[0.05-0.31]) and age > 5 years (aOR=0.03, 95%CI[0.01-0.08]). Stratified by age group, anaemia was significantly associated with stunting in children less than 5 years (aOR=3.1 95%CI[1.4 – 6.8]), with, sickle cell disorders (aOR=3.5 95%CI [1.4 – 9.0]), alpha-thalassemia (or=2.4 95%CI[1.1–5.3]) and stunting (aOR=3.6 95%CI [1.6–8.2]) for children above 5 years. No association was found between G6PD deficiency, intestinal worm carriage and children’s gender.

**Conclusion:**

Malaria parasitaemia, stunting and haemoglobin genetic disorders represented the major causes of anaemia among study participants. Anaemia control in this area could be achieved by developing integrated interventions targeting both malaria and malnutrition.

## Background

Anaemia (haemoglobin < 11 g/dl) is a major public health problem affecting 1.62 billion people globally [1]. Africa and Asia are the most affected regions with more than 85% of the absolute anaemia burden [1]. Children and women of reproductive age are most at risk, with global anaemia prevalence estimates of 47% in children younger than 5 years, 42% in pregnant woman and 30% in non-pregnant woman aged 15 – 49 years. [1,2] Causes of anaemia can be broadly classified into decreased erythrocyte production or increased loss of erythrocytes through increased destruction (haemolysis) or blood loss or both [2]. In developing countries, anaemia aetiologies are multi-factorial, and infectious diseases, genetic haemoglobin disorders, as well as malnutrition, can play a major role in anaemia occurrence [3].

Infectious diseases such as intestinal parasite infections contribute to anaemia through impaired absorption and metabolism of iron and other micronutrients or increased nutrient losses. For instance, soil-transmitted helminth infections commonly found in tropical regions are a major cause of anaemia [4,5]. Other parasitic infections such as schistosomiasis are frequent in sub-Saharan countries [6] and can lead to anaemia due to iron deficiency from blood lost from haematuria caused by *Schistosoma haematobium,* or diarrhoea due to *Schistosoma mansoni.* Malaria remains an important cause of morbidity in tropical regions with an estimated 149 to 274 million cases and 539 000 to 906 000 deaths worldwide [7]. *Plasmodium falciparum* is the most pathogenic species and can lead to increased erythrocyte destruction and thus to anaemia [8].

Genetic haemoglobin disorders, which are the consequence from irregular structural or reduced production of the globin chains of haemoglobin, can result in anaemia [2]. Several studies have investigated the distribution and functional consequences of these genetic disorders; while it is clear that heterozygotes (carriers) of the mutations are partially protected against malaria, [9,10] their contribution to the global anaemia burden remains less clear [11] and studies are becoming even more relevant [12] when malaria is declining, as in Senegal, and several other previous high-endemic areas on anaemia [13,14]. Sickle-cell disorders are associated with haemolytic anaemia and an estimated 2.28 individuals per 1000 births worldwide are affected by sickle-cell disorders [15]. Sickle cell disease is the homozygous state for haemoglobin S (HbSS), caused by a mutation in the β-haemoglobin. Sickle cell trait is the heterozygous state of this haemoglobinopathy (mainly HbAS) [12]. Alpha thalassemia is also a genetic haemoglobinopathy highly prevalent in Sub-Saharan Africa, Asia and Melanesia [10]. Normal individuals have duplicate α-genes on each chromosome 16. By contrast, those with heterozygous α+− thalassemia have loss of 1 α-gene, resulting in 3 functional α-genes (−α/αα), whereas homozygotes have only 2 functional α-genes (−α/-α). Heterozygote individuals are characterized by slight haematological changes, whereas homozygote individuals generally have mild microcytic anaemia [16].

Another prevalent genetic disorder in previous and present malarious areas is glucose-6-phosphate-dehydrogenase (G6PD) deficiency. G6PD deficiency is a common chromosome x-linked red blood cell enzymopathy with several polymorphisms arisen from mutations in the G6PD gene [17]. In sub-Saharan Africa, G6PD is essentially a tri-allelic polymorphism. G6PD (B) is the most common allele with normal enzymatic activity; G6PD (A) is associated with a single amino acid substitution at codon (c) 126 where Asn is changed to Asp (N126D), causing around 85% of the normal enzymatic activity. The G6PD (A-) deficiency allele has a single amino acid substitution at c68 from Val to Met (V68M), always in conjunction with the N126D mutation [18]. The G6PD (A-) variant has only around 12% of normal enzymatic activity with frequencies of 5– 25% of the affected population in sub-Saharan Africa [19,20]. Although most individuals with the G6PD (A-) polymorphic variant are asymptomatic, acute haemolytic anaemia can manifest in hetero and homozygote females as well as hemizygote males under oxidative stress of the red blood cells [19]. This condition can be induced by infections, anti-inflammatory agents and chemotherapeutics, including anti-malarials such as primaquine and dapsone [17].

Malnutrition is also known to be a factor associated with anaemia [21]. Restricted access to diverse micronutrients particularly in vulnerable groups living in low-income countries can contribute to malnutrition as well as to anaemia [2].

Overall, in tropical regions anaemia causes present a great diversity. Although anaemia aetiologies are multi-factorial, most studies in malaria endemic areas have been confined to the anaemia associated with malaria or with other single factors [22-25]. In recent years, significant reductions in the incidence of malaria have been reported in several African countries where malaria was previously highly to moderately endemic [13]. A study conducted in Senegal (2010), showed a significant reduction of malaria cases in children who had access to several antimalarial interventions. However, despite the significant reduction of malaria, anaemia prevalence remained high in these communities [14]. It remains thus, unclear why anaemia is still high in communities who had access to high coverage of antimalarial interventions. Therefore, this study was undertaken, to document malaria risks for anaemia as well as other possible risks factors and determinants in an area with declining of malaria pattern.

## Methods

### Study area

The study was conducted in the 8 villages covered by the Bonconto health post, which is located in the Velingara health district in the South-eastern part of Senegal, 500 km from the capital city of Dakar. Mass deworming administration (MDA) program using Mebendazole is being promoted in the area of Bonconto health post with two yearly administrations in children under 5 years. Reports from the health post records showed coverage of mebendazole administration at 95% in December 2010.

### Study design and population

A cross sectional survey was carried out in December 2010 at the end of the malaria transmission season, and 3 weeks after the MDA campaign in the 8 villages covered by the Bonconto health post. Study participants were randomly selected from the list of children who participated to the survey in December, using a random number generator from Excel^TM^ software. Children aged from 1 to 10 years with haemoglobin level below 11 g/dl were considered as anaemic and represented the cases while control participants were eligible if of same age group (1 to 10 years) and their haemoglobin level was at least 11 g/dl.

### Sample size calculation

Among the investigated factors, malaria is known to be a common risk factor associated to anaemia; thus, malaria prevalence was used as the main outcome for sample size estimation. Based on 80% power, 5% significance level, assuming an overall carriage of malaria parasitaemia among non anaemic children in the study area at 22% (*Senegal Malaria indicator survey 2009*), a minimum odds ratio of 2 and a ratio case/control =1, a number of 354 participants (177 cases and 177 controls) was required in this study.

### Data collection

#### Clinical assessment

Each child was examined by a physician prior to a biological assessment which included blood, stool and urine samples. The mother was interviewed directly to determine socio-demographic characteristics as well as the child history of fever, using a standard questionnaire. Symptoms presented by each child at the day of survey as well as data obtained from physical examination and the parent interview were assigned in a case report form (CRF).

#### Anthropometric measurements

Children’s weight and height were measured by a trained field worker. Weight­for­age Z­score was used to denote underweight while height-for-age Z-score was used as an indicator of stunting. The Z­scores were calculated based on the median values of the National Centre for Health Statistics (NCHS) reference population, United States.

#### Laboratory methods

##### Parasites detection

Blood samples were collected using finger prick blood. The first drop was used for thick and thin smear tests for the diagnosis of malaria. Thick and thin smear tests were stained with Giemsa. Parasite density was determined by counting the number of asexual parasites per 200 white blood cells, and calculated per μl using the following formula: numbered parasites × 8000 / 200, assuming a white blood cell count of 8,000 cells per μl. Absence of malaria parasites in 200 high power ocular fields of the thick film was considered as negative.

Fresh stools samples were collected into clean containers. Faecal samples were examined for the detection of intestinal parasites using Ritchie technique. Intestinal parasites were recorded positive by the presence of helminths and/or protozoans in the faeces.

Urine samples were collected into clean containers between 10 to 14 h. To determine the presence of *Schistosoma haematobium* eggs in urine, a filtration method using polycarbamate nucleopore filters was used. One aliquot of 10 ml of urine sample was filtered and placed on a slide and examined using light microscopy.

### Haemoglobin concentration determination

A drop of finger prick blood was drawn into a microcuvette for Hb determination (g/dl) using a HemoCue machine (HemoCue Hb 201®). Anaemia was defined as Hb concentration below 11 g/dl.

Due to some logistical constraints, samples for haemoglobin concentration, malaria parasite investigation and haemoglobin genetic disorders investigation, were collected in December 2010, while stool and urine samples were collected later in January two weeks after the first samples drawn.

### Identification of human genetic polymorphisms in study participants

#### Samples and DNA extraction

Blood samples were collected on filter paper from 352 children who participated in the survey in December 2010. DNA was extracted from segments of bloodspots on filter paper in 96 well plate formats by chelex-100 methods as described elsewhere [26]. Haemoglobin A, S, C and the G6PD B, A and A-, were determined using PCR followed by a sequence-specific oligonucleotide probe enzyme-linked immunosorbent assay (SSOP-ELISA) while alpha-thalassemia was detected by PCR alone [20-27].

#### Polymerase chain reaction (PCR) conditions

Primers were used to amplify a 352 base-pair (bp) fragment of the G6PD gene covering the mutation site at codon 68 [27]. The haemoglobin gene was amplified by primers described by Modiano et al. [9] to produce a 358 bp fragment covering the mutation sites at codon 6 and 26. Primers were biotinylated at the 5′end by the supplier (MWG Biotech, Ebersberg, Germany) [20]. For alpha thalassemia, primers were used to produce bands of 2200 + 1900 bp representing the African α-globin variants [28].

The PCR conditions for both the G6PD and HbB gene were similar to the conditions described in [20]. The reactions were performed in 96 well PCR plates on a DNA thermal cycler. Alpha thalassemia PCR products were visualized by electrophoresis on a 0.75% agarose gel.

#### SSOP-ELISA

The procedures have been described in [20], in brief; oligonucleotide probes of 18 bases covering the SNP of interest were used where the 3′ end was conjugated with digoxigenin. Amplified PCR products were diluted 1:2 in dH_2_O, denatured at 95°C for 5 minutes. Two μl of the PCR products were added to streptavidin coated (1μl/ml PBS) ELISA plates containing washing buffer (PBS with 0.05% Tween 20) and incubated for one hour at room temperature. The bound PCR products were incubated with 8 nM of probe in tetra-methyl ammonium chloride (TMAC) solution at 53°C with shaking for an hour. TMAC washing temperatures were set to 62°C for haemoglobin probes and 65°C for the G6PD probes. The plates were incubated with peroxidase conjugated antidigoxigenin antibodies (Roche Diagnostics) 1:1000 in washing buffer at room temperature for one hour, thereafter visualized by o-phenylene-diamine (OPD). The reaction was stopped with H_2_SO_4_ before measuring the optical density at 492 nm. Between each step, the ELISA plates were washed three times in washing buffer.

#### Data management and data analysis

Data were entered in Excel ^TM^ software and analysed using STATA 11^TM^ software. To assess the nutritional status, data were transferred into Epi Info. The Z­scores for weight­for­age (underweight) and height­for­age (stunting) were derived using Epinut Anthropometry (Epi Info sowfware). Children who had z scores below −2 standard deviations (SD) of the NCHS median reference population were considered to be malnourished. G6PD deficiency (G6PD A-) was defined as hemizygotes males and/or homozygote females. Sickle cell disease was represented by the homozygote state for haemoglobin S (HBSS) while the heterozygote state (HBAS, HBAC), represented sickle cell trait. Alpha-thalassemic children were represented by hetero and homozygote children. For categorical data, percentage was used to assess the frequency of each outcome. For continuous data, mean and standard deviation were used to describe normally distributed variables, median and range for other data. Characteristics of all children included in the study were tabulated by study group. Proportions were compared using chi square test or Fisher exact test where appropriated (univariate analysis); significance level of the different tests was set on 0.05, two sided. A stepwise logistic regression was done for the determination of risk factors possibly associated with anaemia.

#### Ethical considerations

This study was approved by the Senegalese National Ethical Committee (Conseil National de Recherche en Santé). Informed consent was obtained from parents or children guardian at the time of survey.

## Results

### Study participants characteristics

A total number of 352 children less than 10 years of age were enrolled in the study (176 cases and 176 controls). Children less than 5 years represented 69% in the anaemic group and 26% in the non-anaemic group (p=0.001). The proportion of boys was 60% in the anaemic group and 53% in the non-anaemic group (p=0.13). A proportion of 50% in both groups had access to seasonal intermittent preventive treatment with SP-AQ. Bed-net ownership in the anaemic group represented 100%, while that for the non-anaemic group represented 98%. (p=0.08) (Table 1).

**Table 1:** Socio demographic characteristics of study participants

**Anaemic group (176)**			**Non anaemic group (n=176)**		
**Characteristics**	**Number (%)**	**95%CI**	**Number (%)**	**95%CI**	***p value***
**Age group**					
Under 5	121 (68.75)	[57.04-82.14]	46 (26.14)	[19.13-34.86]	0.001
[5 - 10 years]	54 (30.68)	[23.05-40.03]	130 (73.86)	[61.71-87.70]	0.001
Female	69 (39.20)	[30.50-49.61]	83 (47.16)	[37.56-58.46]	0.13
Male	106 (60.27)	[49.31-72.84]	93 (52.84)	[42.65-64.73]	0.16
Children within household					
[1 – 3]	85 (48.29)	[38.58-59.72]	58 (32.95)	[25.02-42.60]	0.003
[4 -5]	52 (29.54)	[22.06-38.74]	68 (38.64)	[30.00-48.98]	0.07
>5	38 (21.59)	[15.28-21.63]	50 (28.41)	[21.08-37.45]	0.13
Birth order					
[1 -3]	106 (60.22)	[49.31-72.84]	109 (61.93)	[50.85-74.71]	0.74
[4 – 5]	42 (23.86)	[17.19-32.25]	40 (22.73)	[16.23-30.95]	0.80
>5	27 (15.34)	[10.11-22.32]	27 (15.34)	[10.11-22.32]	1
Access to IPTc with SP-AQ	88 (50)	[40.10-61.60]	88 (50)	[41.10-61.60]	1
Bed-net ownership	176 (100)	[85.7-116]	173 (98.30)	[84.19-114]	0.08

Among the 176 children in the anaemic group, 122 (69.3%) were less than five years old while the remaining proportion (30.6%) had an age ranged from 5 to 10 years. Overall, mild anaemia was predominant in children above the age of 5 years (63%). Moderate anaemia represented a proportion of 48.4% in children less than 5 years and 33.3% in children with an age from 5 to 10 years (p=0.06). Few children were found with severe anaemia: 6.5% among under 5 years and 3.7% in children above 5 years (p=0.68) (Table 2).

**Table 2:** Frequency and severity of anaemia among cases stratified by age group

	**Under 5years (N=122)**		**[5 - 10years] (N=54)**		**p value**
	**n (%)**	**95%CI**	**n (%)**	**95%CI**	
Mild anaemia (Hb<11g/dl)	55 (45.1)	[34.0-58.7]	34 (63.0)	[43.6-87.9]	0.03
Moderate anaemia (Hb <9g/dl)	59 (48.4)	[36.8-62.4]	18 (33.3)	[19.7-52.7]	0.06
Severe anaemia (Hb<7g/dl)	08 (6.5)	[2.8-12.9]	02 (3.7)	[0.4-13.4]	0.68

### Parasitic infections among study participants

*Plasmodium falciparum* parasites were identified in 11 children (3.12%). Prevalence of *P. falciparum* was evaluated at 5.11% and 2.14%, respectively in anaemic children and non-anaemic children (p=0.03).

A total number of 97 children (27%) were found with at least one intestinal parasite. The identified intestinal parasites were represented by: *Giardia intestinalis* (12%), *Entameaba coli* (17%), *Strongyloides stercoralis* (0.6%). Hookworms, *Schistosoma haematobium* and *Schistosoma mansoni* were not found (Table 3).

**Table 3:** Overall prevalence of parasitic infections, erythrocytes polymorphisms and malnutrition among study participants

**Outcome**	**No / Total No (%)**	**95%CI**
**Malaria parasitaemia**	11/352 (3.12)	[1.56-5.59]
**Intestinal parasite carriage**	97/352 (27.56)	[22.35-33.62]
Isolated intestinal parasites		
*Giardia intestinalis*	43/352 (12.22)	[8.84-16.45]
*Entameaba coli*	61/352 (17.33)	[13.25-22.26]
*Strongyloides stercoralis*	02/352 (0.57)	[0.06-2.05]
**Genetic disorders**		
G6PD A	07/352 (1.99)	[0.79-4.09]
G6PD A-	03/352 (0.85)	[0.17-2.49]
Alpha-thalassemia (Hetero+Homozygote)	78/352 (22.16)	[17.51-27.65]
Heterozygote (αα/-α)	62/352 (17.61)	[13.50-22.58]
Homozygote (−α/-α)	16/352 (4.55)	[2.60-7.38]
Sickle cells disorders (HBSS + HBAS +HbAC)	45 (12.78)	[9.32-17.10]
Sickle cell disease (HBSS)	10/352 (2.84)	[1.36-5.22]
Sickle cell trait (HBAS, C)	33/352 (9.38)	[6.45-13.17]
**Malnutrition**		
Stunting	136/352 (38.64)	[32.42-45.70]
Underweight	91/352 (25.85)	[20.81-31.74]
Wasting	33/352 (9.38)	[6.45-13.17]

A total number of 42 children (24%) in the anaemic group were found with at least one intestinal parasite compared to 55 children (31%) in the non-anaemic group (p=0.12). The prevalence of isolated intestinal parasites was similar between the two study groups. (Table 4).

**Table 4:** Prevalence of parasitic infections, erythrocytes polymorphisms and malnutrition in each study arm

**Anaemic group (n=176)**			**Non anaemic group (n=176)**		
**Characteristics**	**Number (%)**	**95%CI**	**Number (%)**	**95%CI**	**p value**
Malaria parasitaemia	9 (5.11)	[2.34-9.70]	2 (1.14)	[0.13-4.10]	0.03
Intestinal parasite carriage	42 (23.86)	[17.19-32.26]	55 (31.25)	[23.54-40.67]	0.12
Parasite species					
*Giardia intestinalis*	21 (11.93)	[7.39-18.23]	22 (12.50)	[7.83-19.92]	0.87
*Entameaba coli*	26 (14.77)	[9.65-21.64]	35 (18.89)	[13.81-27,65]	0.20
*Strongyloides stercoralis*	00	--	02 (1.14)	[0.13-4.11]	0.15
Sickle cells					
AS patients	22 (12.57)	[7.83-19.92]	11 (6.25)	[3.11-11.18]	0.04
SS patients	07 (3.97)	[1.59-8.19]	03 (1.70)	[0.35-4.98]	0.19
AC patients	01 (0.57)	[0.01-3.16]	01 (0.57)	[0.01-3.16]	1
Alpha thalassaemia *(Hetero and homozygotes)*	48 (27.27)	[20.11-36.16]	30 (17.05)	[11.50-24.33]	0.02
Heterozygotes Alpha thalassemia	37 (21.02)	[14.80-28.97]	25 (14.20)	[9.19-20.97]	0.09
Homozygotes Alpha thalassemia	11 (6.25)	[3.11-11.18]	05 (2.84)	[0.92-6.23]	0.12
G6PD (B)	148 (84.09)	[71.08-98.78]	134(76.14)	[63.79-90.17]	0.06
G6PD (A)	3 (1.70)	[0.35-4.98]	4 (2.27)	[0.61-5.81]	0.70
G6PD (A-)	3 (1.70)	[0.35-4.98]	00	--	0.08
Stunting	91 (51.70)	[41.62-63.48]	45 (25.57)	[18.64-34.21]	0.001
Underweight	61 (34.66)	[26.51-44.52]	30 (17.05)	[11.50-24.33]	0.001
Wasting	15 (8.52)	[4.77-14.06]	18 (10.23)	[6.06-16.16]	0.58

### Erythrocytes polymorphism among study participants

The overall prevalence of sickle cell trait (HbAS and HbAC) was evaluated at 9.94% (35 children) where the majority (33 children) were HbAS while two children were found with HbAC. Children with sickle disease (HbSS) represented 2.8% (10 children).-). Sickle cell disease (HbSS) represented 3.9% (7 children) in the anaemic group and 1.7% (3 children) in the non-anaemic group (p=0.19). A total number of 23 anaemic (13%) and 12 non-anaemic children (6.8%) were found with sickle cell trait (HbAS or HBAC), respectively (p=0.04). Heterozygote alpha-thalassemia (αα/-α) was found in 62 children (17.6%) and homozygote alpha-thalassemia was detected in 16 children (4.5%). Thus, in total the prevalence of alpha-thalassemia (hetero and homozygote) was 22.1% (78 children). Alpha-thalassemia (Hetero and homozygotes) was detected in 48 anaemic children (27.2%) and in 30 non-anaemic children (17%) (p=0.02). Heterozygote alpha-thalassemia was prevalent in 21% of anaemic children and 14% of non-anaemic children (p=0.09). Homozygote alpha-thalassemia represented 6.2% in the anaemic group (11 children) and 2.8% (5 children) in the non-anaemic group (p=0.12) (Table 4).

The G6PD A was detected in 7 children (2%) while 3 children (0.8%) were caring the G6PD A-. Comparison between the two study groups showed a prevalence of G6PD (A) at 1.7% (3 children) in the anaemic group and 2.2% (4 children) in the non-anaemic group (p=0.70). The G6PD (A-) was detected in 3 anaemic children (1.7%) while no child in the non-anaemic group was found with G6PD (A).

### Nutritional status

The overall prevalence of stunting among study participants was evaluated at 38.64% (136 children) while underweight and wasting represented 25.85% and 9.38%, respectively (Table 3). Among anaemic children, stunting represented 52% (91 children), whereas only half, 26% (45 children) in the non-anaemic group (p=0.001). Prevalence of underweight was 34% (61 children) in the anaemic group versus 17% (30 children) in the non-anaemic group (p=0.001). Wasting was found in a proportion of 8% and 10% respectively in anaemic and non-anaemic children (p=0.58) (Table 4).

### Factors associated with anaemia among study participants

In a logistic regression analysis, anaemia was in overall significantly associated with malaria parasitaemia (aOR=5.2, 95%CI [1.1-28.4]), Sickle cell disorders (aOR=2.8, 95%CI [1.3-6.3]), Alpha-thalassemia (aOR=1.8, 95%CI [1.2-3.3]), and stunting (aOR=3.4, 95%CI [1.9-5.9]. Age group was also significantly associated with anaemia, adjusted OR at 0.1 for children aged from 2 to 4 years and 0.03 for children above 5 years of age. (Table 5) Stratified by age group, anaemia was significantly associated with stunting in children less than 5 years (aOR=3.1 95%CI [1.4 – 6.8]). In children aged from 5 to 10 years, the risk factors significantly associated with anaemia were represented by: stunting (aOR=3.6 95%CI [1.6 – 8.2]), sickle cell disorders (aOR=3.5 95%CI [1.4 – 9.0]), alpha-thallasemia (aOR=2.4 95%CI [1.1 – 5.3]) (Table 6). The study did not find any statistically significant association between intestinal parasites and anaemia, nor between anaemia and co-infection with more than one intestinal parasite.

**Table 5:** Distribution of factors associated with anaemia among study participants

	**Frequency**		**Multivariate analysis**	
	**Anaemic group (n=176)**	**Non anaemic group (n=176)**	**aOR (95%CI)**	**p value**
**Variables**				
**Gender**				
Female	69 (39.43%)	83 (47.16%)	*Reference*	
Male	106 (60.57)	93 (52.84%)	1.37 [0.82-2.31]	0.22
**Age group**				
1year	57 (32.76%)	8 (4.55%)	*Reference*	
[2–4 years]	89 (51.15%)	73 (45.48%)	0.13 [0.05-0.31]	0.001
[5 – 10 years]	28 (16.09%)	95 (53.98)	0.03 [0.01-0.08]	0.001
**Access to IPTc drug (SP-AQ)**				
No	87 (49.71%)	88 (50%)	*Reference*	
Yes	88 (50.29%)	88 (50%)	0.78 [0.45-1.33]	
**Stunting**				
No	84 (48%)	131 (74%)	*Reference*	
Yes	91 (52%)	45 (25.57%)	3.37 [1.93-5.88]	0.001
**Malaria Parasitaemia**				
No	166 (94.86%)	174 (98.86%)	*Reference*	
Yes	9 (5.14%)	2 (1.14%)	5.23 [1.1-28.48]	0.04
**Sickle cell disorders**				
No	145 (82.86%)	161 (91.48%)	*Reference*	
Yes	30 (17.14%)	15 (8.52%)	2.89 [1.32-6.34]	0.008
**Alpha Thalassemia**				
No	128 (73.14%9	146 (82.95%)	*Reference*	
Yes	47 (28.86%)	30 (17.05%)	1.82 [1.2-3.35]	0.04

aOR: adjusted odds ratio. IPTc: Intermittent Preventive Treatment in children. SP-AQ: Sulfadoxine-Pyrimethamine. Goodness-of-fit-test: Hosmer-Lemeshow, Chi2 (8df)=5.07, p=0.75.

**Table 6:** Risk factors significantly associated with anaemia in children less than 5 years and children aged from 5 to 10 years at the Bonconto health post, Senegal

**Risk Factors**	**aOR**	**95%CI**	**p value**
Children under 5 years (n=122)*			
Stunting	3.1	[1.4 - 6.8]	0.004
**Children aged 5 to 10 years (n=54)** †			
Stunting	3.6	[1.6 - 8.2]	0.002
Sickle cell disorders	3.5	[1.4 - 9.0]	0.009
Alpha-thalassemia	2.4	[1.1 - 5.3]	0.026

*Analysis of risk factors associated to anaemia among children under 5 years was adjusted on the following variables: P. falciparum infection, G6PD deficiency, Sickle cell disorders, Alpha-thallassemia, Access to IPTc drug (SP-AQ), gender. The number of anaemic children in this age group was = 122. Goodness of fit test: Hosmer-Lemeshow, chi(8df)=0.99, p=0.99. † Analysis of risk factors associated to anaemia among children aged from 5 to 10 years was adjusted on the following variables: P. falciparum infection, G6PD deficiency, Access to IPTc drug (SP-AQ), gender. The number of anaemic children was= 54. Goodness of fit test: Hosmer-Lemeshow, chi(6df)=4.22, p=0.64.

## Discussion

Although anaemia aetiologies are often multi-factorial, most studies have been confined to the anaemia associated with malaria or other individual factors [22-25]. This study investigated several factors potentially associated to anaemia among children less than 10 years living in rural area in Senegal. In this area, recently, a randomized trial prior to the present case control study was conducted to assess the impact on malaria of combining home based management and intermittent preventive treatment [14]. Thus, children involved in the case control study, had access to several antimalarial interventions and furthermore, the area has a high coverage of impregnated bed nets.

The high coverage of antimalarial interventions in the study area, and the fact that the study was conducted at the end of the malaria transmission season, could explain the low prevalence of malaria parasitaemia among the study participants (3.1%). Still, malaria prevalence was higher in anaemic children compared to non anaemic children and was closely associated to anaemia. Thus, scaling up antimalarial interventions at community level such as early case detection and treatment, intermittent preventive treatment as well as long lasting insecticide treated nets, may be a step towards malaria elimination, but will as well reduce the burden of anaemia. However, other aetiologies need to be considered for an effective and integrated control of anaemia.

Intestinal protozoan infections (*Giardia intestinalis, Entameaba coli*) were the predominant parasites isolated from stool samples among the study participants. No significant association between these parasites and anaemia was observed. Despite that, *G. intestinalis* may induce diarrhoea and mal-absorption syndrome, which can lead to vitamin B12 deficiency as well as iron deficient anaemia [29]. The low prevalence of intestinal helminthic infections could be explained as a result of regular mass deworming administration program in the study area. Indeed, two mass de-worming campaigns using mebendazole (June and December every year) were undertaken in the study period as part of a national policy aiming at improving child survival. As Giardia is still prevalent in the area of Bonconto and it has the potential to induce diarrhoea and mal-absorption syndrome, this parasite could be targeted by public health programs. Thus, shifting from mebendazole to albendazole, which may be used against giardiasis, could contribute to further reduce it in children.

Sickle cell disorders (HbAS, HbAC and HbSS) among study participants were frequent with an overall prevalence at 12.8% which are consistent with other findings; Mbodj et *al.* in 2003, reported a prevalence of HbAS and HbSS in the general population in Senegal at 11.1% [30], in line with Diop et *al.* findings in 2005, of 10% [31]. Alpha-talassaemia (hetero and homozygote) was more prevalent among study participants (22%). In 2006, Nabias et *al.* reported a similar prevalence of alpha-thalassemia (24%) in a cohort of children aged from 2 to 10 years living in a rural area of Senegal [32]. Sickle cell disorders and alpha-thalassemia were strongly associated with anaemia in this study and thus may play a major role in the global burden of anaemia in developing countries [2]. Indeed, as child survival improves, and malaria is decreasing in many African countries, [13] inherited haemoglobin disorders could become an increasingly important disease burden and cause of anaemia in the future [33]*i.e*. beneficial balanced polymorphisms may become unbalanced. However, lack of adequate diagnostic tools in poor African settings can in this context, impact negatively on the investigation of anaemia related to genetic haemoglobin disorders. Therefore, there is a need to strengthen health systems diagnostic capacities in African settings, in order to optimize the investigation of genetic haemoglobin disorders and their consequences such as anaemia.

In this study, a low prevalence of G6PD deficiency was found: G6PD (A-) patients represented a proportion of 0.85%. This is much less than the 12.3% reported in another study from Senegal [31]. G6PD deficiency is usually asymptomatic but can lead to haemolytic anaemia under oxidative stress [19]. Although no significant association was found between the prevalence of G6PD deficiency and anaemia in this study, this genetic disorder was more prevalent in anaemic children.

Malnutrition was frequent in this study with an overall prevalence of 38% for stunting, 25% for underweight and 9% for wasting. Stunting was closely associated to anaemia. Malnutrition is known to be a leading factor to anaemia [34]. An inadequate intake of macro and micronutrients, or intestinal mal-absorption induced or increased by intestinal parasites infections, can play through iron and folate deficiency, a well-documented role in chronic anaemia pathophysiology [5].

Overall, malaria parasitaemia, stunting and some human genetic disorders represented the major causes of anaemia among study participants. Malaria and stunting can be controlled individually or together by developing integrated interventions targeting malaria and malnutrition. A study conducted at the Bonconto health post showed that home based management of malaria can be combined with intermittent preventive treatment, both delivered by community health workers with a significant impact on malaria and anaemia [14]. In addition micronutrient supplementation could be combined to home based management of malaria and IPTc in order to further reduce malnutrition and anaemia prevalence in children less than 10 years. However, studies are needed in such area in order to document the feasibility as well as the impact of such strategies on child survival. Some studies have shown that vitamin A and iron supplementation (alone), may actually negatively impact child survival and malaria outcome [35,36]. The potential benefit of iron supplementation coupled with effective malaria controls for children living in malaria-endemic regions, need to be investigated.

### Study limitations

This study has provided useful data for anaemia prevention and control. The study was designed as a case control study. Although selection of controls was restricted to children aged from 1 to 10 years, children less than 5 years were predominant in the anaemic group. To control for confounding induced by age group, multivariate analysis was done with adjustment by age group among other factors.

Iron deficiency has been shown to be an important factor in the pathophysiology of anaemia. Details on iron deficiency and other micronutrients deficiencies (which have not have not been evaluated in this study), would add to revealing anaemia aetiologies in this part of Senegal. The role of iron deficiency in anaemia occurrence varies according to age groups. Indeed, a study conducted recently in Ivory Coast, found a high prevalence of iron deficiency in infants, without however any significant association with anaemia among infants [37]. Among preschool children, living in areas with high prevalence of soil transmitted helminthic infection (STH) such as hookworm, iron deficiency can play a major role in anaemia occurrence [37]. In this study, no child was found with hookworm infection, neither *Schistosoma* which could contribute to iron deficiency anaemia.

## Conclusion

Malaria parasitaemia, stunting and haemoglobin genetic disorders represented the major causes of anaemia among children between 1 and 10 years participating in this study. Anaemia control in this area could be achieved by developing integrated interventions, targeting both malaria and malnutrition.

## Competing interests

The authors have no conflicts of interest concerning the work reported in this paper.

## Authors’ contributions

RCT, CTN, PM, MA, ICB, OG conceived and designed the study. RT, MN and KS collected the data. RT, MA and HHH were responsible for execution and analysis of molecular data on human genetic polymorphisms. RT analysed the data. RT, CTN, PM, ICB, OG, BF, HHH, MA wrote the manuscript. All authors read and approved the final manuscript.
